# Tandem RNA binding sites induce self-association of the stress granule marker protein TIA-1

**DOI:** 10.1093/nar/gkab080

**Published:** 2021-02-24

**Authors:** Fionna E Loughlin, Danella L West, Menachem J Gunzburg, Saboora Waris, Simon A Crawford, Matthew C J Wilce, Jacqueline A Wilce

**Affiliations:** Monash Biomedicine Discovery Institute and Department of Biochemistry and Molecular Biology, Monash University, Victoria 3800, Australia; Monash Biomedicine Discovery Institute and Department of Biochemistry and Molecular Biology, Monash University, Victoria 3800, Australia; Monash Institute of Pharmaceutical Sciences, Monash University, Victoria 3052, Australia; Monash Biomedicine Discovery Institute and Department of Biochemistry and Molecular Biology, Monash University, Victoria 3800, Australia; Ramaciotti Centre For Cryo Electron Microscopy, Monash University, Victoria 3800, Australia; Monash Biomedicine Discovery Institute and Department of Biochemistry and Molecular Biology, Monash University, Victoria 3800, Australia; Monash Biomedicine Discovery Institute and Department of Biochemistry and Molecular Biology, Monash University, Victoria 3800, Australia

## Abstract

TIA-1 is an RNA-binding protein that sequesters target RNA into stress granules under conditions of cellular stress. Promotion of stress granule formation by TIA-1 depends upon self-association of its prion-like domain that facilitates liquid-liquid phase separation and is thought to be enhanced via RNA binding. However, the mechanisms underlying the influence of RNA on TIA-1 self-association have not been previously demonstrated. Here we have investigated the self-associating properties of full-length TIA-1 in the presence of designed and native TIA-1 nucleic acid binding sites *in vitro*, monitoring phase separation, fibril formation and shape. We show that single stranded RNA and DNA induce liquid-liquid phase separation of TIA-1 in a multisite, sequence-specific manner and also efficiently promote formation of amyloid-like fibrils. Although RNA binding to a single site induces a small conformational change in TIA-1, this alone does not enhance phase separation of TIA-1. Tandem binding sites are required to enhance phase separation of TIA-1 and this is finely tuned by the protein:binding site stoichiometry rather than nucleic acid length. Native tandem TIA-1 binding sites within the 3′ UTR of p53 mRNA also efficiently enhance phase separation of TIA-1 and thus may potentially act as potent nucleation sites for stress granule assembly.

## Graphical Abstract

**Graphical Abstract:**
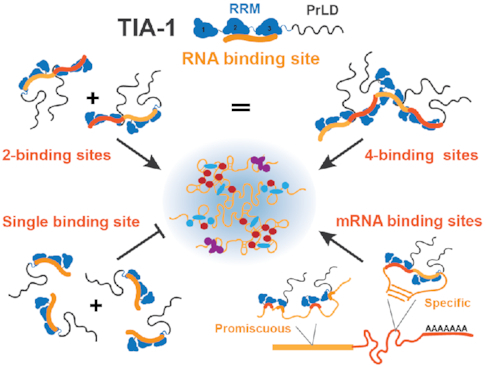
Self-association of TIA-1 is induced by tandem (specific or promiscuous) RNA binding sites in a manner that is finely tuned by the protein:RNA binding site ratio rather than RNA length.

## INTRODUCTION

Stress granules (SGs) are non-membranous assemblies of protein and mRNA that form reversibly in the cytosol of cells. They serve as a protective cellular mechanism under stresses such as heat, oxidative, osmotic or nutrient-limitation. Under such conditions, translation initiation is blocked and, as polysomes complete elongation, newly exposed mRNAs and associated proteins condense to form SGs (1). There, the mRNA is held in a translationally silent state and is largely protected from degradation. This allows the cell to prioritize translation of protective proteins, such as heat shock proteins, that enable cell survival. SGs persist in the cell for several hours, until the stress event is resolved, after which they are disassembled and translation of mRNA is able to recommence. Persistent SGs that do not disassemble are associated with neurodegenerative diseases, potentially acting as crucibles for protein aggregates that are toxic to the cell (2,3). There has therefore been an extensive effort to understand the molecular forces that drive SG formation as well as their progression to toxic aggregate.

The molecular forces that drive SG formation include a core network of protein–RNA, protein–protein and RNA–RNA interactions that collectively result in an effective phase partition from the aqueous surrounds (4–6). Newly exposed messenger RNAs condense via liquid–liquid phase separation (LLPS) through base pair and stacking interactions and through interactions with RNA-binding proteins. Condensation of RNA-binding proteins is commonly mediated by weak multivalent protein–protein interactions, occurring between intrinsically disordered domains (7). In particular, SGs are enriched with proteins containing prion-like domains (PrLDs) that are a subclass of intrinsically disordered domains rich in Asn/Gln/Tyr/Gly residues (8). These regions have a propensity to undergo LLPS via interactions involving distributed tyrosines. They can also form labile kinked β-sheet interactions as well as more stable β-zippers which, over time, can propagate to form amyloid like structures (9,10). Thus, functional SG proteins can potentially propagate via protein–protein interactions to form irreversible aggregates associated with neurodegenerative disease (3,11).

What is less well known is the way in which protein–RNA and protein-protein interactions act together in the formation of biomolecular condensates such as stress granules. In particular, the role of RNA and the way in which it regulates phase separation of RNA binding proteins is not well understood. Intriguingly, RNA can both enhance and suppress phase separation of a number of RNA binding proteins that are present within RNA granules (12–15). The influence of RNA on phase separation of RNA binding proteins is reported to be mediated through specific (16,17) or promiscuous interactions (11,13) between the RNA and protein. Upon interaction RNA may act as a scaffold, increasing the local concentration of RNA binding proteins, or regulate protein function by inducing intramolecular conformational changes (18,19). Conversley, high concentrations of RNA have been shown to suppress phase separation of RNA binding proteins in the nucleus (15). Understanding the mechanistic details underlying the way in which RNA impacts phase separation of RNA binding proteins is important for gaining insight into the fundamental mechanisms of stress granule formation and dissolution.

**Figure 1.**
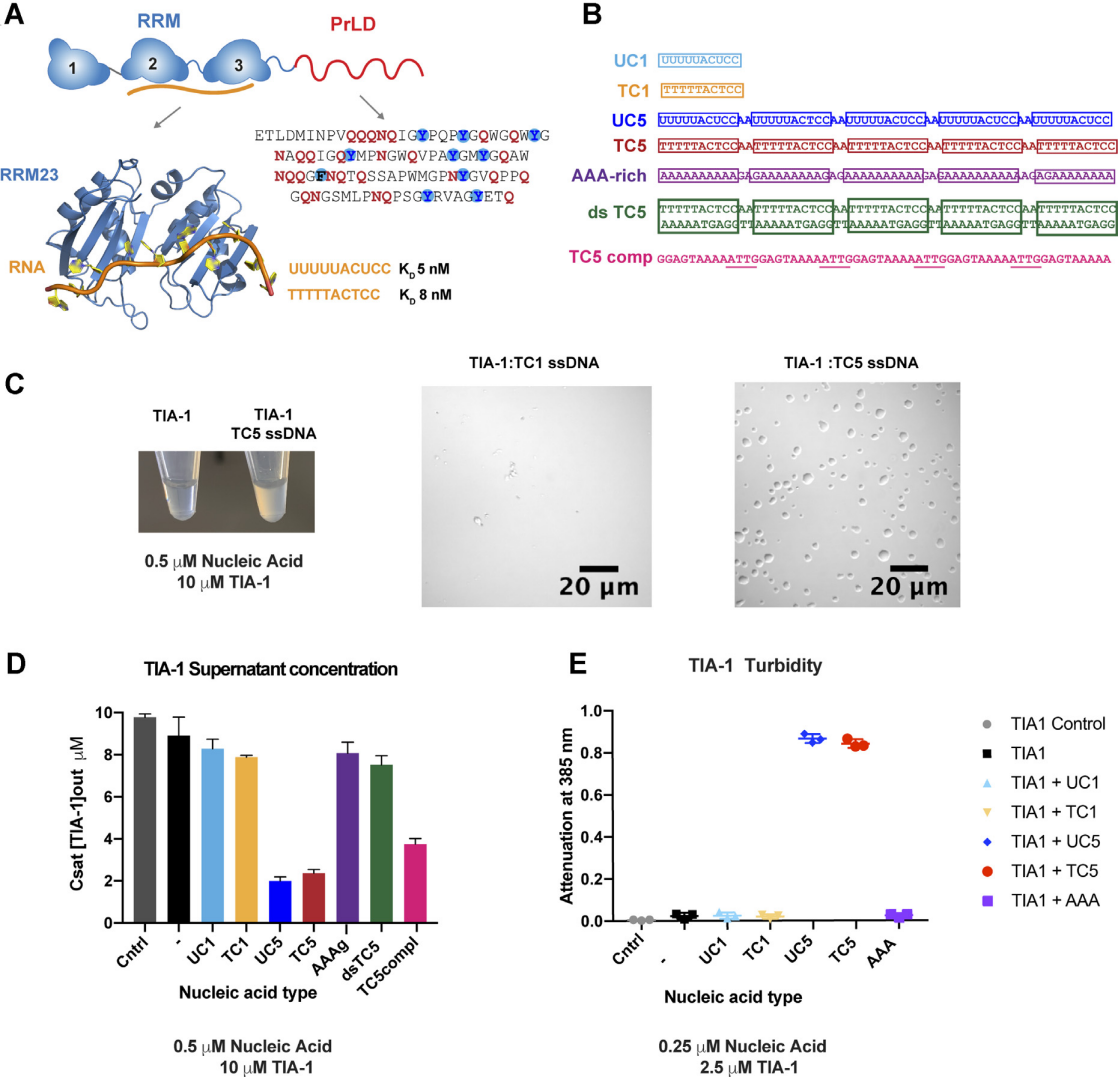
Sequence specific multisite RNA and DNA induce liquid-liquid phase separation of TIA-1. (**A**) Schematic of TIA-1 domain composition (*top*), model of nucleic acid binding by RRM23 (*left*) and sequence of TIA-1 Prion-Like Domain showing characteristic Gln/Asn in red and Phe/Tyr in blue circles. (**B**) Nucleic acid sequences used in this study highlighting TIA-1 target sites in boxes. (**C**) Addition of ssDNA induces opalescence in TIA-1 sample (*left*) DIC microscopy of TIA-1 (10 μM) in the presence of single site (TC1) or multisite (TC5) nucleic acid (0.5 μM) in aggregation buffer (20 mM HEPES pH 7, 50 mM NaCl, 40 mM Arginine) (*right*). (**D**) The concentration of TIA-1 protein remaining in dispersed supernatant solution (*C*_sat_) after phase separation of TIA-1 (10 μM) instigated by addition of nucleic acids (0.5 μM). Cntrl represents TIA-1 diluted in size exclusion buffer in which TIA-1 remains soluble (20 mM sodium phosphate, 60 mM KCl, 0.5 M arginine–HCl, 1 mM MgCl_2_, 2 mM DTT, 0.5 mM EDTA, pH 7.0). (−) Indicates TIA-1 diluted in aggregation buffer in the absence of nucleic acids. (**E**) Turbidity assay of TIA-1 (2.5 μM) alone or in the presence of nucleic acid (0.25 μM). Assays show three replicates and error bars representing S.D.

The current study investigates the influence of RNA on the self-association and phase separation of a major stress granule protein, T-cell intracellular antigen protein (TIA-1). TIA-1 is prototypical RNA-binding protein that comprises three N-terminal RNA-recognition motifs (RRMs) and a C-terminal PrLD (Figure 1A). TIA-1 regulates target mRNA translation in the cytosol and, under conditions of stress, condenses via the intrinsically disordered PrLD to form SGs (20). TIA-1 PrLD alone has been shown to undergo LLPS *in vitro* and is recruited to SGs *in vivo* (21,22). Furthermore, mutations in TIA-1 PrLD that are associated with neurodegenerative disease and multisystem proteinopathy have been shown to increase the aggregation potential of TIA-1 *in vitro* and delayed SG disassembly *in vivo* (23). However, the RRMs are also essential for bone fide SG formation by TIA-1. When full-length TIA-1 is overexpressed in cells there is spontaneous formation of SGs, but overexpression of TIA-1 PrLD lacking its RRMs results only in the formation of microaggregate (22). This suggests that RNA interactions are important for the formation of SG potentially acting to nucleate TIA-1 driven SG formation. Hence the current study was undertaken to examine the influence of RNA binding on TIA-1 condensation.

In this study we use a series of synthetic designed and native TIA-1 RNA binding sequences to investigate the influence of RNA binding on TIA-1 phase separation *in vitro*. Based on previous work (24–26), we used a high affinity U/C rich target sequence that TIA-1 recognises via RRMs 2 and 3. Using this sequence, we show that multiple high affinity TIA-1 binding sites in single stranded RNA or DNA induce TIA-1 phase separation and enhance amyloid fibril formation over time. In contrast, a single binding site does not facilitate TIA-1 phase separation or fibril formation, although it does induce a detectable conformational change. Upon varying the length of the oligonucleotide we show that tandem binding sites are sufficient to induce TIA-1 condensation independent of length and upon varying the ratio of TIA-1 to oligonucleotide binding site we show that condensation can be finely tuned. Furthermore, extending the study to a native target, we show that tandem TIA-1 binding sites in TIA-1 regulated 3′ UTR of p53 (27) promotes TIA-1 condensation whereas a single TIA-1 binding site within the same 3′ UTR does not. Together these results suggest that tandem TIA-1 RNA binding sites act as a sequence specific seeds able to induce local TIA-1 condensation on larger RNAs. This suggests that SG formation in cells may be triggered by newly exposed mRNA sequences containing tandem pyrimidine rich sites that act as nucleation sites for TIA-1 condensation.

## MATERIALS AND METHODS

### TIA-1 protein expression and purification

TIA-1 protein was prepared from plasmid pETM-11 encoding the short isoform of human TIA-1 (P31483-2). TIA-1 expression was performed in BL21 pLysS *Escherichia coli* cells and protein expression was induced at OD 0.6–8 with 0.5 mM IPTG followed by 4 h at 25 °C. Cells were resuspended in lysis buffer (50 mM sodium phosphate, 300 mM NaCl, 50 mM arginine, 2 mM MgCl_2_, 2 mM imidazole, pH 7.0) with the addition of DNase I and cOmplete TM EDTA-free protease inhibitor cocktail (Roche). Cells were lysed using a cell homogenizer (Avestin), following which the NaCl concentration was adjusted to 1 M and the lysate clarified by centrifugation at 48 000 g for 45 min. Soluble TIA-1 protein was bound to Co-TALON metal affinity resin (Takara), washed with wash buffer (50 mM sodium phosphate, 300 mM NaCl, 15 mM imidazole, 10% glycerol, pH 7.0) and eluted in elution buffer (50 mM sodium phosphate, 300 mM NaCl, 0.5 M arginine–HCl, 150 mM imidazole, pH 7.0) to which 2 mM DTT was added. Fractions were dialysed into TEV cleavage buffer (50 mM sodium phosphate, 300 mM NaCl, 0.5 M arginine–HCl, 10% glycerol, pH 7.0) and the His-tag was cleaved from TIA-1 with TEV protease overnight at 4 °C. The His-tag was separated from TIA-1 using Ni-NTA resin in TEV cleavage buffer. Untagged TIA-1 protein was further purified by size exclusion chromatography in size exclusion buffer (20 mM sodium phosphate, 60 mM KCl, 0.5 M arginine–HCl, 1 mM MgCl_2_, 2 mM DTT, 0.5 mM EDTA, pH 7). Pure fractions were concentrated to a maximum of 100 μM, filtered and stored in aliquots at -80 °C. Protein was quantified using the theoretical molar extinction coefficient 80 330 M^−1^ cm^−1^. The *A*_260/280_ ratio of nucleic acid free TIA-1 protein was 0.62. Protein purity was analysed by SDS-PAGE and ESI Mass spectrometry confirming a mass of 42 307.9 Da (theoretical mass 42 308.4 Da).

### Oligonucleotides

Synthetic DNA and RNA oligonucleotides (listed in Supplementary Table S1) were synthesized and HPLC purified commercially (IDT) and further analysed on denaturing 16% acrylamide gels visualized with SYBR Gold nucleic acid gel stain. Double stranded DNA ‘dsTC5’ was prepared by annealing ‘TC5’ and ‘TC5 complement’ and purifying dsDNA by size exclusion chromatography. Oligonucleotides were quantified using molar extinction coefficients supplied by IDT.

### Yeast RNA

Torula yeast RNA type VI (Sigma No. R6625) was resuspended at 15 mg/ml in 20 mM HEPES pH 7.2, 50 mM NaCl, then desalted using PD-10 desalting column (Cytivia) and quantitated by absorbance *A*_260nm_ 1 = 40 μg/ml ssRNA.

### p53 3’ UTR RNA

DNA templates of mouse p53 3′ UTR (NM 011640.3 nt1412–1437) were generated by PCR from synthetic geneblocks incorporating a T7 promoter. Sequences of mutated TIA-1 target sites were based on those reported in (27). RNAs were prepared using *in vitro* RNA polymerase transcription reactions followed by phenol chloroform extraction, buffer exchanged into 5 mM HEPES pH 7.0 and folded by heating up to 95 °C for 10 min followed by snap cooling on ice prior to use.

### Measurement of C_sat_

To quantify the *C*_sat_ of TIA-1 alone and in the presence of nucleic acids, we used the centrifugation approach (23). Purified, filtered TIA-1 in size exclusion buffer (20 mM sodium phosphate, 60 mM KCl, 0.5 M arginine–HCl, 1 mM MgCl_2_, 2 mM DTT, 0.5 mM EDTA, pH 7.0) was diluted to 10 μM alone or in the presence of 0.5 μM nucleic acid in aggregation buffer (20 mM HEPES, 50 mM NaCl pH 7.2 including residual 40 mM arginine–HCl). The concentration of nucleic acid was kept low in order to minimize interference with protein quantitation. Control samples included dilution of TIA-1 to 10 μM into size exclusion buffer in which TIA-1 remains soluble and dilution of nucleic acids alone to 0.25 μM in aggregation buffer. Triplicate samples of 100 μl were incubated at room temperature (∼22 °C) or 4 °C for 10 min in LoBind 1.5 ml tubes (Eppendorf) followed by centrifugation at 13 000 × g for 10 min at the respective temperature. The supernatant was analysed by a nanodrop spectrophotometer at 280 and 260 nm. In addition, SDS-PAGE was used to detect changes in TIA-1 protein concentration and Urea-PAGE stained with SYBR Gold to detect nucleic acids in initial and supernatant samples. For TIA-1:nucleic acid samples in which nucleic acid remained in the supernatant as ascertained by Urea-PAGE and *A*_260/280_ ratios (A-rich, dsTC5), *A*_280_ measurements were corrected for contribution from soluble nucleic acids, to prevent overestimation of TIA-1 concentration (Supplementary Tables S2 and S3). Final *C*_sat_ concentrations of TIA-1 were based on corrected *A*_280_ measurements using the molar extinction coefficient of TIA-1. Assay included three replicates with error bars representing S.D.

### Turbidity measurements

To quantify phase separation of TIA-1 at lower protein concentrations and higher nucleic acid: protein ratios than possible in *C*_sat_ measurements, a turbidity assay was used. TIA-1 protein in size exclusion buffer was diluted either alone, or in the presence of nucleic acids in aggregation buffer. In general, TIA-1 protein was diluted to 2.5 μM in the presence of 0.25 μM nucleic acids unless otherwise stated. As controls, nucleic acids alone were diluted in aggregation buffer. Triplicate samples of 150 μl were incubated for 10 min at room temperature in 96-well μClear® non-binding black plates (Greiner) at room temperature then analysed immediately at 385 nm using a CLARIOstar plate reader (BMG Labtech). Assays included three replicates with error bars representing S.D.

### DIC microscopy

Samples of TIA-1 with or without oligonucleotides were prepared fresh as per samples used in *C*_sat_ or Turbidity measurements and imaged within 10–20 min of mixing. An additional sample was taken directly from the ThT assay plate at the end point of the assay. A 10 μl aliquot of each sample was spotted onto a glass microscope slide and covered with 0.17 mm HP glass coverslip (Zeiss). Solutions were imaged at room temperature with differential interference contrast on a FV1000 Confocal Microscope (Olympus) or DMi8 (Leica) with a 60× objective and processed using FIJI (28).

### Thioflavin T assays

Thioflavin-T (ThT) fluorescence assays were used to monitor ThT positive aggregate formation of TIA-1 alone and in the presence of nucleic acids. Samples were prepared as per turbidity assays in aggregation buffer with the addition of 5 μM ThT in 96-well non-binding black plates (Greiner) but equilibrated to 30 °C for 30 min prior to measurements commencing. Samples were then agitated through orbital shaking 500 r.p.m. at 30 °C over 12–16 h and ThT fluorescence monitored with excitation at 425 nm and fluorescence emission at 485 nm using a CLARIOstar plate reader (BMG Labtech). Measurements were baseline corrected using ThT fluorescence of aggregations buffer/ThT alone. An exception included the ThT assay of TIA-1 in the presence of increasing TC5 concentrations in which measurements were baseline corrected against for ThT fluorescence of DNA alone. Assays included three to four replicates with error bars representing S.E.M.

### Transmission electron microscopy

To analyse aggregates present at the endpoint of the ThT assay, the final samples were resuspended and a 5 μl aliquot was placed on the surface of a charged copper grid coated with Fromvar. Samples were stained with 2% uranyl acetate solution and excess stain was removed using filter paper. Images were recorded using a Joel 1400 Plus 120 keV TEM at Ramaciotti Centre for Cryo-Electron Microscopy Monash University.

### Fluorescent anisotropy assays

Fluorescence anisotropy (FA) assays were performed in 96-well non-binding black plates (Greiner) using a PHERAstar plate reader (BMG Labtech). 5′-fluorescein labelled ‘TC1’ ssDNA oligonucleotide (1 nM) was incubated with either (i) TIA-1 RRM23 protein in 10 mM HEPES pH 7.2, 50 mM NaCl, 1 mM DTT or (ii) TIA-1 full length protein in size exclusion buffer in which TIA-1 is monomeric (20 mM sodium phosphate, 60 mM KCl, 0.5 M arginine–HCl, 1 mM MgCl_2_, 2 mM DTT, 0.5 mM EDTA, pH 7). Data were analysed using PRISM using a 1:1 specific site model to estimate the *K*_D_.

### Small angle X-ray scattering data collection and analysis

TIA-1 protein alone or incubated with oligonucleotide ‘UC1’ or ‘TC1’ (1:1.2 ratio) was extensively dialysed against TIA-1 buffer (20 mM sodium phosphate, 60 mM KCl, 0.5 M arginine-HCl, 1 mM MgCl_2_, 2 mM DTT, 0.5 mM EDTA, pH 7.0) and concentration of protein and nucleic acid verified. Samples were centrifuged for 13 200 × g for 15 min before data collection. SAXS data were collected in static format at the SAX-WAXS beamline at the Australian Synchrotron. Samples at 0.37, 0.75, 1.45 and 2.9 mg/ml of protein (equivalent to 8.7, 17.2, 34 and 68 μM TIA-1) were loaded in 80 μl volumes in a 96-well V bottom plate (Axygen) and covered with silicon sealing mat. SAXS data reduction was performed using scatterBrain developed at the Australian Synchrotron. Further processing including averaging, buffer subtraction, and [P(r)] derivation were performed using the PRIMUS module of ATSAS 2.8 (29). The ensemble optimization method 2.0 (EOM 2.0) (30,31) was used to examine the degree flexibility and conformational mobility of TIA-1 in the presence and absence of DNA or RNA. EOM was applied to each SAXS data set to select the minimum ensemble of structures from a pool of 50 000 that best fit the SAXS data. Structures used for modelling TIA-1 RRMs included PDB 5O2V (32), 2MJN (33) and 1CVJ as previously used to model TIA-1 RRM2/3 (34). The three RRMs were allowed to move independently to one another and the PrLD was fully flexible.

## RESULTS

### TIA-1 phase separates and aggregates *in vitro* in a concentration dependent manner

To analyse the effect of RNA on condensation of TIA-1 *in vitro*, we first investigated conditions under which TIA-1 protein alone undergoes LLPS and fibril formation. Full-length recombinant monodispersed TIA-1 was prepared in a stabilised form in buffer conditions that included 500 mM arginine (Supplementary Figure S1A&B). To detect the phase separation of TIA-1 protein we measured the concentration of TIA-1 remaining dispersed in solution—known as the saturation concentration (*C*_sat_) of TIA-1 (35). Phase separation of TIA-1 was instigated by simultaneous dilution of the protein and arginine into HEPES buffer (pH 7) at either 4 °C or room temperature. As a control, TIA-1 was also diluted into high concentration arginine buffer in which TIA-1 remains soluble. The protein-rich heavy-phase was sedimented by centrifugation and the protein depleted light-phase of soluble TIA-1 was quantified. Over the concentration range of 2.5–10 μM the *C*_sat_ of TIA-1 at 4 °C was consistently observed at ∼1.7–2 μM concentration (Supplementary Figure S1C left). In contrast, at ∼22 °C most of the TIA-1 at each concentration remained in the light-phase (Supplementary Figure S1C right). These results indicate a spontaneous and temperature dependent phase separation of TIA-1 protein.

In order to analyse potential fibril formation by TIA-1 we employed a Thioflavin T (ThT) assay which is highly sensitive for detection of amyloid fibrils and less so for LLPS. Following dilution of arginine and TIA-1 into HEPES buffer containing ThT dye, ThT fluorescence was monitored for 16 h, while shaking at 30 °C. An increase in ThT fluorescence was observed following a short lag time indicative of β-sheet rich structures forming in a concentration dependent manner (Supplementary Figure S1D). At a TIA-1 concentration of 10 μM, the lag time was short (approximately 50 minutes from the start of shaking) before the fluorescence rapidly increased to a maximum value, whereas at 2.5 μM the ThT fluorescence remained low for the duration of the experiment. These results are consistent with published reports showing that tagged fusion TIA-1 protein undergoes phase separation and, over a longer time can form amyloid fibrils (23,36,37). Thus, experimental conditions were established in which TIA-1 protein alone does not show LLPS or apparent fibril formation and therefore we could test the enhancing effect of RNA.

### Design of oligonucleotides with multi TIA-1 specific target sites

To determine whether the intrinsic self-association propensity of TIA-1 is affected by RNA binding and/or whether RNA acts as a scaffold for TIA-1 condensation we designed a series of oligonucleotides based on a previously delineated high affinity binding site for TIA-1 (34). RRM2 of TIA-1 has a stringent selectivity for U rich sequences, whereas RRM3 also contributes to recognising C rich TIA-1 RNA targets (26). In contrast, RRM1 does not robustly bind RNA (33). We thus used a uracil–cytosine rich RNA sequence (5′-UUUUUACUCC-3′ henceforth referred to as ‘UC’) that is bound with high affinity by TIA-1 RRM23 (*K*_D_ = 5 nM). A ssDNA version of this site (‘TC’) is also bound with high affinity (*K*_D_ = 8 nM, Figure 1A) (34). This allowed us to probe the effect of the addition of RNA or DNA comprising either one or several concatenated UC/TC target sites on TIA-1 condensation. We designed RNA sequences with either 1 (UC1) or 5 (UC5) target sites separated by ‘AA’ dinucleotide (Supplementary Table S1 and Figure 1B). Concatenated UC sites were chosen, rather than stretches of U10 that are also bound by TIA-1 (24,38), due to the well-defined orientation of RRM23 on the UC motif and lower propensity of such sequences to form non-canonical base pair structures.

### Multi-site nucleic acid enhances liquid-liquid phase separation of TIA-1

We first determined the effect of nucleic acid on LLPS of full-length TIA-1. For this we used single and multiple TIA-1 nucleic acid binding site oligonucleotides (UC1 and UC5 RNA, TC1 and TC5 DNA, as well as double stranded TC5 and A-rich DNA as negative controls) combined with excess TIA-1 protein (Figure 1B). We observed changes in solution opalescence, the appearance of droplets using DIC microscopy and, as a more quantitative measures of phase-separation, the concentration of protein remaining dissolved in solution (*C*_sat_) and the turbidity of the solution (attenuation of 385 nm light). Dilution of TIA-1 into HEPES buffer at room temperature resulted in a clear solution, as would be expected at this temperature. The TIA-1 solution also remained clear in the presence of TC1. In contrast, the presence of TC5 in the TIA-1 solution resulted in spontaneous formation of an opalescent character indicative of robust phase separation (Figure 1C). DIC microscopy of these same samples showed a small number of droplets formed by TIA-1 in the presence of TC1, but formation of numerous large liquid-liquid phase separated droplets in the presence of TC5 (Figure 1C, Supplementary Figure S1E). To quantify the effect of nucleic acid addition on phase separation by TIA-1 we first measured the saturation concentration (*C*_sat_) of 10 μM TIA-1 at room temperature in the presence or absence of oligonucleotide. In this assay TIA-1 protein diluted into buffer including 500 mM arginine in which TIA-1 remains soluble showed no protein depletion *C*_sat_ ∼10 μM (Figure 1D). Similarly, TIA-1 diluted in HEPES buffer alone showed minimal protein depletion with a *C*_sat_ of ∼9 μM. The presence of UC1 or TC1 had no significant effect on the *C*_sat_, nor did the A-rich non-cognate ssDNA or a dsDNA version of TC5. In contrast, the presence of UC5 and TC5 reduced *C*_sat_ to ∼2 μM indicating a significant enhancement of phase separation. Surprisingly the sequence complement of TC5 ssDNA also effectively induced phase separation of TIA-1 with a *C*_sat_ of ∼3.5 μM. On closer inspection, the complement of the ‘AA’ linker residues generate four ‘ATT’ sites which resemble the AUU TIA-1 target sub-motif in A-U rich elements (AREs) and thus may serve as sub-optimal TIA-1 binding sites (24). To ensure *C*_sat_ measurements represent a bone fide decrease in TIA-1 protein concentration in the supernatant, TIA-1 protein from total or supernatant was also inspected by SDS-PAGE (Supplementary Figure S1F). As an additional, more sensitive quantification of phase separation, at protein concentrations closer to estimated cellular levels (∼1.4 μM in HeLa cells) (39), we used a turbidity assay that measures the formation of light scattering aggregates suspended in solution. We observed no increase in turbidity of 2.5 μM TIA-1 alone or in the presence of UC1, TC1 or the A-rich sequences (Figure 1E), In contrast we observed a robust increase in turbidity in the presence of UC5 or TC5 (Figure 1E, Supplementary Figure S1G). This was far greater than the turbidity of solutions of oligonucleotide alone (Supplementary Figure S1H). Together these results demonstrate that addition of nucleic acids with multiple, accessible, sequence specific TIA-1 binding sites enhance LLPS of TIA-1.

### Multi-site nucleic acid enhances fibril formation by TIA-1

**Figure 2.**
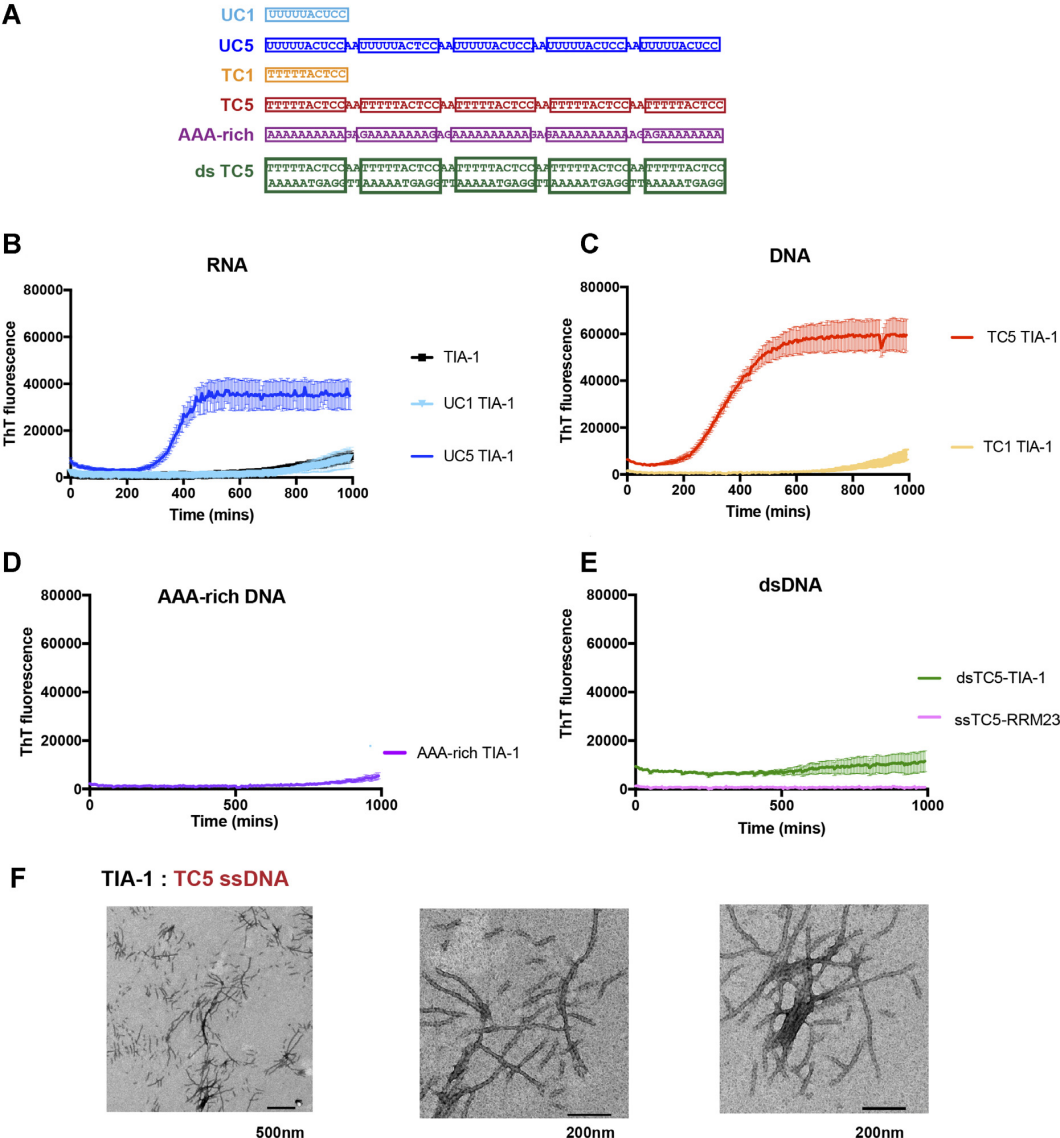
Sequence specific multisite RNA and DNA induces fibril formation of TIA-1. Fibril formation of TIA-1 monitored by Thioflavin-T fluorescence alone and in the presence nucleic acids. (**A**) Nucleic acid sequences sequences used in this study highlighting TIA-1 target sites in boxes. (**B**) TIA-1 protein alone and with RNA containing single (TC1) or multisite (TC5) TIA-1 binding sites, (**C**) DNA containing single (TC1) or five (TC5) TIA-1 binding sites, (**D**) Size matched A-rich DNA without TIA-1 binding sites, (**E**) double stranded DNA including five TIA-1 binding sites in the top strand (dsDNA TC5) and TIA-1-RRM23 domains with DNA containing five TIA-1 binding sites (ssDNA TC5). (**F**) Representative TEM images of fibrils induced with DNA containing five TIA-1 binding sites (TC5). TIA-1 2.5 μM, nucleic acid 0.25 μM in 20 mM HEPES pH 7, 50 mM NaCl, 40 mM arginine at 30 °C. Thioflavin T assays show four replicates with error bars representing S.E.M.

Nucleic acid has been shown to increase polymerization of RNA binding PrLD protein FUS into hydrogels and fibrillar aggregates (40,41). We considered whether this could also be the case for TIA-1 and whether it could occur through sequence specific TIA-1 binding sites. Thus, we next analysed the effect of nucleic acids on aggregation by TIA-1 over time using the amyloidophylic dye thioflavin T (ThT) (Figure 2A). TIA-1 was again diluted to 2.5 μM alone or in the presence of RNA and DNA oligonucleotides in the same manner as used for turbidity assays but then monitored by ThT fluorescence overnight whilst shaking. The presence of UC1 had no effect on TIA-1 Tht-positive aggregation, with the ThT fluorescence showing no increase until after 12 h, similar to TIA-1 alone (Figure 2B). In contrast, the presence of UC5 resulted in a dramatic increase in ThT fluorescence, after a lag time of ∼6 h (Figure 2B). Equivalent observations were made for TC1 and TC5 ssDNA (Figure 2C). In contrast, neither the A-rich ssDNA sequence of equivalent length nor the dsDNA version of TC5 effected the ThT-positive aggregation of TIA-1 (Figure 2D and E). Oligonucleotides alone showed minimal ThT fluorescence at these concentrations (Supplementary Figure S2A–E). Furthermore, a delayed addition of TC5 to TIA-1 resulted in a delay in onset of ThT-positive aggregation (Supplementary Figure S2F) confirming oligonucleotide nucleated aggregation. These aggregates were resistant to SDS detergent indicating they were irreversible (data not shown). Finally, a TIA-1 construct comprising RRMs 2 and 3 (RRM23) alone did not give rise to ThT fluorescence even in the presence of TC5 (Figure 2E) showing that, under these conditions, ThT-positive aggregation of TIA-1 is driven by PrLD interactions in agreement with cellular studies (22). Thus, the same oligonucleotides that drive phase separation also facilitate potential fibril formation.

To characterise the morphology of the nucleic acid induced ThT positive TIA-1 aggregates under the experimental conditions of the assay, samples taken at the ThT assay endpoint were visualized by TEM. These showed the formation of amyloid-like fibrils with a range of lengths from 100 to 1000 nm occasionally clumped or clustered (Figure 2F). Such structures were not present in samples that did not display ThT fluorescence (Supplementary Figure S2G). This is consistent with the ThT assay reflecting the formation of extended β-sheet rich fibrillar aggregates. Although these assays were designed to observe β-sheet rich fibril formation, the final solution was also analysed by DIC microscopy. This revealed the presence of phase separated TIA-1 rich droplets present in addition to the fibrils observed with TEM suggesting that not all the TIA-1 protein converts to amyloid fibrils and that phase separated droplets and fibrils can coexist (Supplementary Figure S2H). From our current data, it is not possible to ascertain whether the amyloid-like fibrils form within phase separated droplets, or independently.

These experiments demonstrate that UC5 and TC5 nucleic acids promote both TIA-1 phase separation and fibril formation due to the presence of TIA-1 binding sites and that the requirement is for sites that are accessible and not buried in secondary structure. Since single site binding-targets (UC1 or TC1) did not induce condensation under the assay conditions, it suggests that the observed condensation induced by UC5 and TC5 occurs through a scaffolding effect by placing multiple TIA-1 molecules in close proximity.

### Conformational change of TIA-1 upon oligonucleotide binding in solution

Although single binding site UC1 and TC1 oligonucleotides had no observable effect on the condensation of TIA-1 in our assays, it did not rule out the possibility that RNA binding by TIA-1 could cause a conformational change impacting its ability to self-associate. In previous SAXS studies, TIA-1 RRM123 has been shown, to be formed of globular domains linked by flexible linkers which, on binding RNA, become more compact (32,34,42). The TIA-1 PrLD is assumed to be intrinsically disordered, but the global conformation adopted in the presence of the RRM domains is unknown. Biochemical evidence showing that TIA-1 RRM1 enhances the interaction between TIA-1 PrLD and spliceosome protein U1C suggests that TIA-1 may exist with the C-terminal PrLD interacting with the globular RRM1 (43). Disruption of this interaction could potentially avail the PrLD for intermolecular-association, similarly to what has been proposed for FUS upon RNA interactions (37). We therefore sought to analyse the overall conformation of TIA-1 in apo and oligonucleotide bound forms.

**Figure 3.**
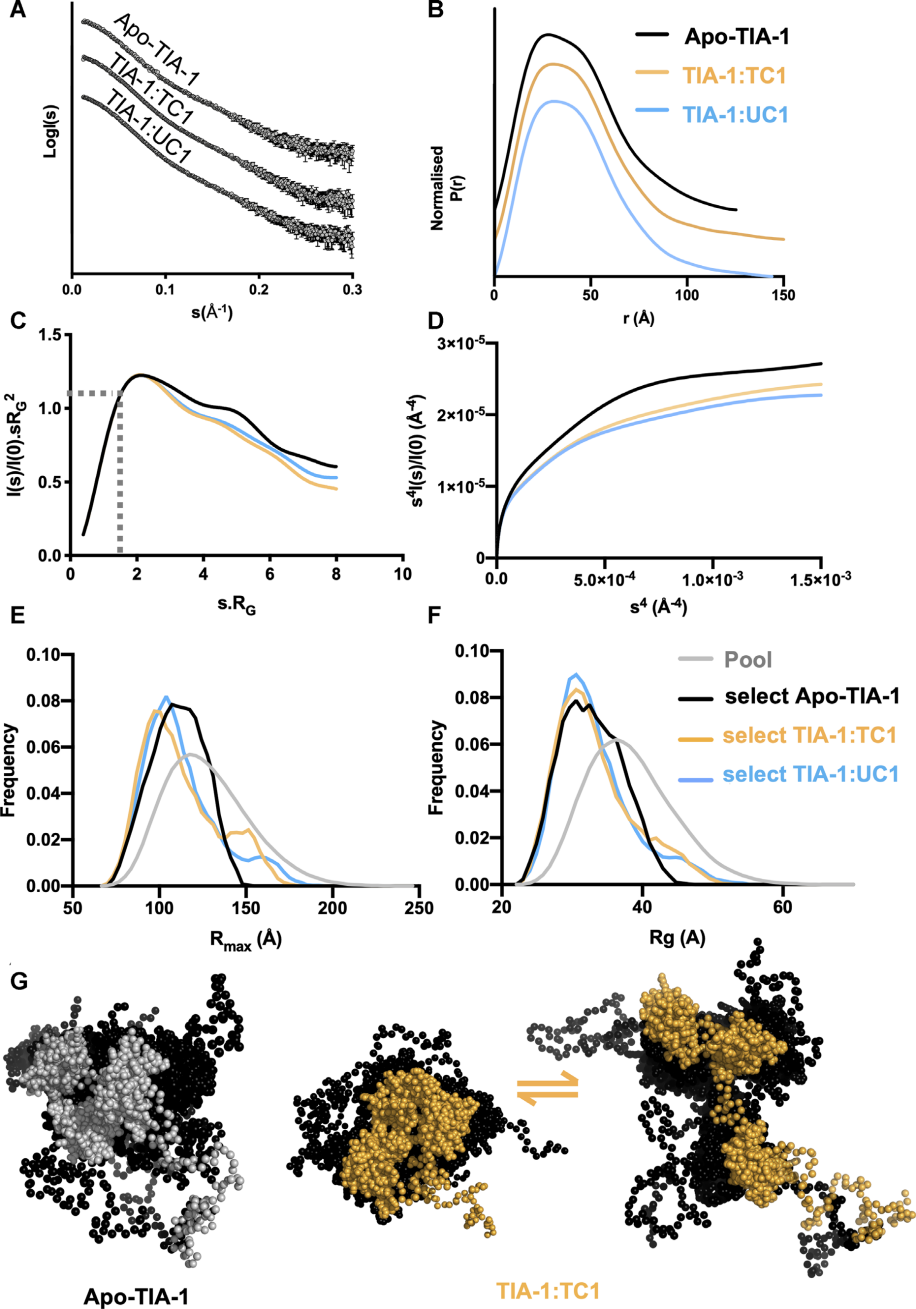
SAXS analysis shows slight conformational change of TIA-1 on binding ssRNA UC1 and ssDNA TC1. (**A**) Merged scattering profiles obtained by plotting the log of scattering intensity [log *I*(*s*)] as a function of forward scattering vector in the units of Å^−1^ and represented by grey circles along with black error bars showing the mean ± SD. (**B**) Pairwise distribution [*P*(*r*)] profiles obtained from scattering data by plotting *P*(*r*) as a function of *r* in the units of Å: TIA-1 (*black*), TIA-1:TC1 (*yellow*), and TIA-1:UC1 (*blue*). (**C**) Smoothed dimensionless Kratky plot obtained from the scattering data by plotting *I*(*s*)/*I*(0).*sR*_g_^2^ versus *sR*_g_^2^. Also indicated is the intersection of *I*(s)/*I*(0).*sR*_g_^2^ = 1.1 with $\sqrt {3\ }$*sR*_g_. (**D**) Porod–Debye profile plot obtained from the scattering data by plotting *s*^4^*I*(*s*)/*I*(0) versus *s*^4^. (**E**, **F**) Ensemble optimization methods of analysis of SAXS data showing *R*_g_ and *R*_max_ distributions from a pool of 10 000 structures (*grey*) or selected ensemble which together best fit the scattering data. (**G**) Overlayed families of TIA-1 structures selected from the ensemble, showing globular RRMs and unstructured PrLD depicted as spheres. Apo-TIA-1 is represented by one family, whereas TIA-1 in complex with oligonucleotide is represented by a combination of a more compact and a more elongated family of structures.

We analysed the conformation of TIA-1 full-length protein in the free state and bound to UC1 and TC1 using SAXS. We used solution conditions that included 500 mM arginine required for mono-dispersed soluble TIA-1 protein and shown not to abolish oligonucleotide binding (Supplementary Figure S3A). Scattering curves were obtained across a concentration range from 0.37, to 2.9 mg/mlL with Guinier derived *R*_g_ values showing good consistency at the lower concentrations (Supplementary Figure S3B and C). The merged data are shown in Figure 3A. The *P*(*r*) plot for apo-TIA-1 shows an asymmetrical profile with a slight shoulder and an *R*_max_ value of about 127 Å (Figure 3B and Table 1). In the case of TIA-1 in complex with UC1 or TC1 the scattering curves were similar to each other but differed slightly from that of apo-TIA-1. The *P*(*r*) plots were slightly less featured and indicative of longer *R*_max_ values of 144 and 151 Å. Interestingly, previous studies have shown that TIA-1 RRM123 (i.e. TIA-1 in the absence of the PrLD) has an *R*_max_ of 100 Å that contracts to 80 Å upon oligonucleotide binding (32,33,42). i.e. In all three of these previous studies there is a clear adoption of a more compact arrangement of the TIA1 RRM domains upon forming a complex with RNA. Thus, assuming that the RRM domains within full-length TIA-1 still adopt a more compact arrangement upon binding RNA, our data reveal that the extended conformation we observed is due to the PrLD under these experimental conditions.

**Table 1.** SAXS derived structural parameters for TIA-1 protein alone or in complex with nucleic acid

Sample	Conc (mg/ml)	*R*_G_ (Guinier) (Å)	*I*_0_ (Guinier)	*R* ^2^	*R*_g_ (PDDF) (Å)	*R*_max_ (Å)	Calculated MW (kDa)	MW from SAXS^a^ (kDa)
**Apo-TIA-1**	2.9–0.37	31.92 ± 0.51	0.051 ± 3.3e-4	0.98	33.56± 0.26	127	42.2	37.7
**TIA-1/TC1**	2.9–0.37	32.41 ±0.57	0.068 ± 2.9e-4	0.97	35.17± 0.33	151	45.2	43.8
**TIA-1/UC1**	2.9–0.37	32.6 ±0.07	0.069 ± 0.0003	0.94	34.57± 0.34	144	45.2	41.9

^a^Calculated by Bayesian Inference according to: Hajizadeh, N.R., Franke, D., Jeffries, C.M. *&* Svergun, D.I. (2018) Consensus Bayesian assessment of protein molecular mass from solution X-ray scattering data. *Sci Rep***8**, 7204.

Note: *R*^2^ = correlation of coefficient.

We also examined the SAXS data for the flexibility information that it can convey. The normalized Kratky plot for apo-TIA-1 shows a plot maximum positioned slightly higher than 1.1 at $\sqrt 3$*sR*_g_ indicating a degree of flexibility (44). This is likewise seen for TIA-1 in complex with oligonucleotide (Figure 3C). The Porod–Debye plot, that allows the rate of decay of the scattering to be easily examined—shows that similar curves are obtained for apo-TIA-1 and TIA-1 in complex with oligonucleotide (Figure 3D). None of the curves approach a plateau, consistent with them all possessing a similar degree of flexibility and the flexibility is greater than that previously reported for TIA-1 RRM123 (42,45). Together this suggests that the PrLD is flexible both before and after oligonucleotide binding by TIA-1 under our experimental conditions.

In order to depict a possible model of TIA-1 consistent with these data we used an ensemble optimisation method (EOM) that is suitable for the modelling of flexible systems (30,31). For this an ensemble of apo-TIA-1 or TIA-1:oligonucleotide structures was generated incorporating the known structures of the folded RRM domains and random structures for the PrLD and linker regions. From this pool a subset of structures was selected which together recapitulated the experimental SAXS scattering curve (Figure 3E and F). The selected structures for apo-TIA-1 spanned a breadth of *R*_max_ and *R*_g_ values, though tended towards the more compact structures within the pool. In the case of TIA-1 bound to oligonucleotide, the majority of selected structures also tended towards the more compact structures, but a small proportion were more elongated. Another, more quantitative measure of the range of structures selected from the pool is the Rflex value – that describes the flexibility of the selected ensemble relative to the pool (30). In all cases we determined lower Rflex values in the selected ensembles (73.27%. 77.25% and 76% for apo-TIA-1, TIA-1:TC1 and TIA-1:UC1 respectively) than that of the pool (82.02%). Figure 3G presents the structures from the pool to illustrate the range of configurations of TIA-1 that were consistent with the SAXS data. Together they show that TIA-1 likely exists as a flexible molecule in equilibrium between more and less compact forms, but upon binding to oligonucleotide, that equilibrium may shift toward more extended forms of the molecules.

### Two TIA-1 binding sites are sufficient to induce TIA-1 condensation

**Figure 4.**
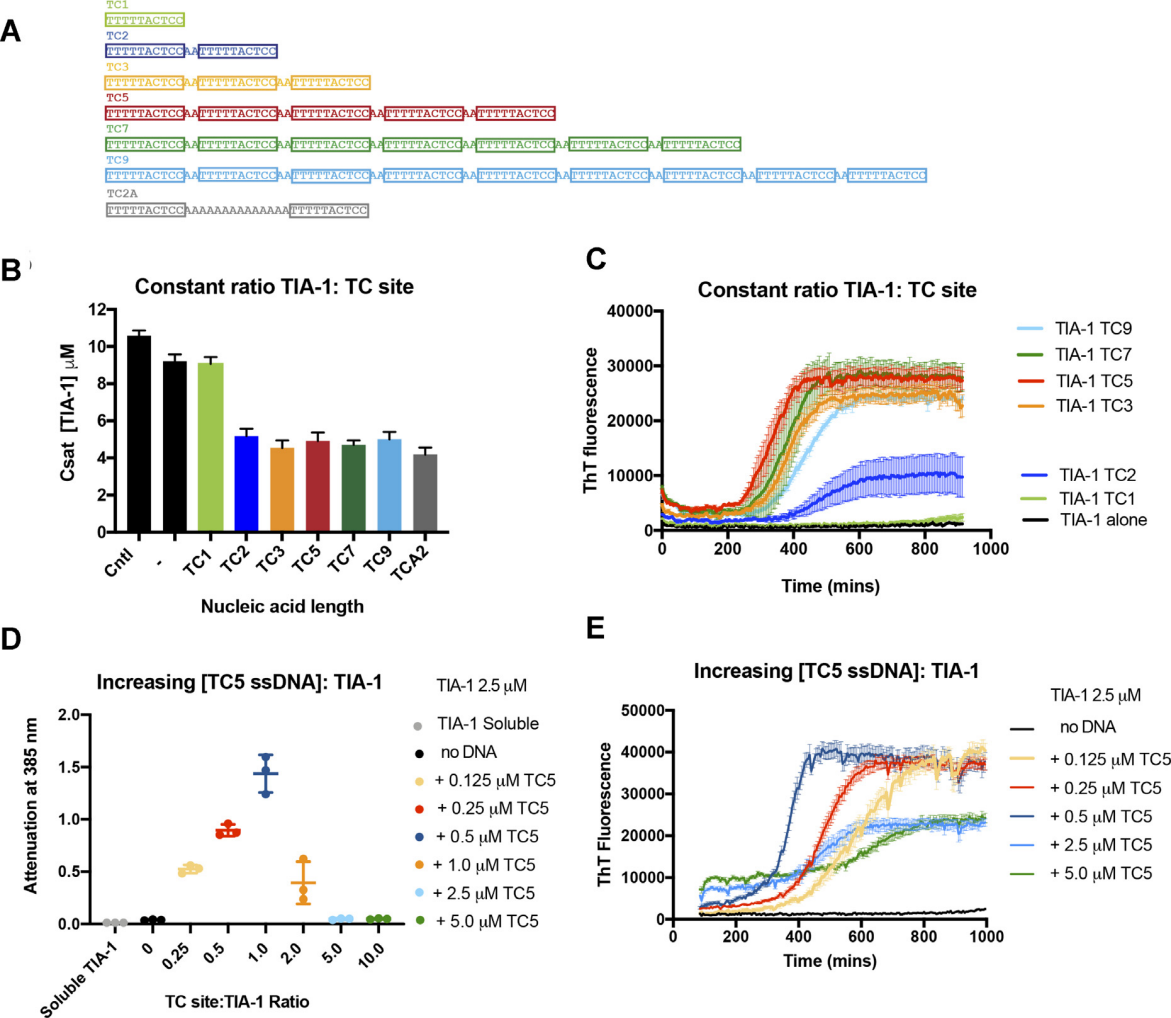
The ratio of TIA-1 protein:binding sites finely tunes liquid-liquid phase separation and fibril formation. (**A**) ssDNA with 1–9 TC binding sites used in this study. (B, C) Measuring the effect of DNA length at constant TC ssDNA:TIA-1 binding site ratio: (**B**) *C*_sat_: Measurement of the concentration of dispersed TIA-1 from 10 μM alone or in the presence of DNA (0.5 μM TC5) with constant TIA-1:TC site ratio of 4:1. (**C**) Thioflavin-T assay of TIA-1 (2.5 μM) showing effect of ssDNA length when adjusted to constant TIA-1:TC site ratio of 2:1. (**D**) Turbidity assay of TIA-1 (2.5 μM) with increasing amount of TC5 (0.125–5 μM). (**E**) Thioflavin-T assay of TIA-1 (2.5 μM) alone (*black*) or in the presence of increasing concentrations of ssDNA TC5 (0.125–5 μM). ThT fluorescence was baseline corrected against ssDNA alone.

Given that the UC5 and TC5 oligonucleotides were highly effective at promoting TIA-1 condensation, we were interested to determine whether the number of TIA-1 binding sites and their proximity to one another would systematically impact TIA-1 condensation. We therefore tested the effects of a series of six ssDNA oligonucleotides comprising 1, 2, 3, 5, 7 or 9 TC DNA sequences separated by ‘AA’ dinucleotides, (Figure 4A). Oligonucleotides were combined with TIA-1 at a 4:1 TIA-1:TC binding site ratio to establish a consistent level of saturation of each binding site. *C*_sat_ was measured to assess the ability of these oligonucletides to induce phase separation (Figure 4B). While TC1 had little effect on the TIA-1 *C*_sat_ compared to TIA-1 alone as expected, TC2 was sufficient for instigating full enhancement of phase separation observed in these assays, inducing a *C*_sat_ value of ∼5 μM. TC3-TC9 instigated a similar effect on TIA-1 phase separation with *C*_sat_ values of between 4.5 and 5.0 μM. Furthermore, when two TC binding sites were separated by a 12 Å spacer (TCA2), a *C*_sat_ of ∼5 μM was obtained showing that TIA-1 binding sites do not have to be immediately adjacent to instigate phase separation (Figure 4B). PAGE analysis confirmed effective depletion of TC2–TC9 nucleic acid from the upper light phase in these experiments consistent with ssDNA:TIA-1 complexes in the condensed phase (Supplementary Figure S4A). Thus strikingly—rather than the longer oligonucleotides showing a greater enhancement effect, TC3–TC9 all showed similar enhancement of TIA-1 aggregation irrespective of length under conditions of excess protein.

Next we analyzed the effect of oligonucleotide length on fibril formation at 2.5 μM with a constant 2:1 TIA-1:TC binding site ratio. ThT assays of TIA-1 fibril formation again showed no effect of TC1. TC2, that comprised two adjacent TC sites, was able to induce TIA-1 fibril formation, but did so inconsistently (showing robust aggregation in two of four replicates Supplementary Figure S4B). TC3, however, robustly induced fibril formation, as did the longer oligonucleotides TC5-TC9 (Figure 4C). In all cases, the ThT fluorescence increased after a lag time of five hours and reached similar plateau levels showing remarkably similar kinetics of fibril formation. Altogether, rather than observing that longer nucleic acids with more binding sites produce a larger enhancement effect, we observed a minimum requirement of 2–3 TC binding sites for nucleic acid to enhance both TIA-1 phase separation and fibril formation and no further enhancement by the longer oligonucleotides.

### Nucleic acid concentration tunes TIA-1 liquid-liquid phase separation and fibril formation

In the course of undertaking these experiments, we observed an interesting effect when we combined TIA-1 and oligonucleotides at a constant protein:DNA molar ratio (rather than a constant protein:TC binding site ratio) such that the relative concentration of TC sites increases with ssDNA length (Supplementary Figure S4C). What we observed in the ThT assay was a short lag time and rapid formation of fibrils induced by TC3, but longer lag times and slower elongation phase of fibril formation as the oligonucleotide lengthened. We reasoned that this effect may be due to effective dilution of free TIA-1 due to the presence of more TC binding sites, and considered whether oligonucleotide concentration and the number of binding sites could tune TIA-1 aggregation in a predictable manner. The ratio of RNA to protein has been shown to tune LLPS of the RNA binding protein FUS with total RNA (13,15,46) and of Arg–Gly–Gly rich peptides with homopolymers (47). We first tested the effect of yeast RNA on TIA-1 phase separation using the turbidity assay over a range of protein:RNA mass ratios (Supplementary Figure S4D). Indeed, under our conditions, this yeast RNA could induce LLPS at low RNA:TIA-1 mass ratios, but not at high RNA ratios further suggesting condensation of TIA-1 may be tuned by nucleic acid concentration.

To directly test the ability of the oligonucleotide concentration with specific binding sites to modulate TIA-1 condensation, we varied the concentration of TC5 between 0.125 and 5 μM and monitored spontaneous phase separation and fibril formation over time of TIA-1 at 2.5 μM (effecting a 0.25–10 TC site:TIA-1 ratio; Figure 4D and E). Since high ssDNA concentration interfere with *C*_sat_ measurements, we used the turbidity assay to measure the effect of TC5 concentration on phase separation. The turbidity of TIA-1 increased with increasing TC5 concentrations until a maximum at 1:1 TIA-1:TC binding site, then reduced to zero in the presence of 5:1 excess TC sites. It should be noted that, TC5 alone showed increasing turbidity with concentration, but not at significant levels compared to experiments involving protein and thus does not underlie the observed phase separation under these conditions (Supplementary Figure S4E). Thus TIA-1 phase separation was found to be highly sensitive to the precise ratio of oligonucleotide binding site:protein. The effect of increasing TC5 ssDNA concentrations with respect to TIA-1 also had a remarkable effect on the on the kinetics of fibril formation over time. We observed more rapid progress towards the fibril elongation phase with increasing oligonucleotide concentration up to a 1:1 ratio of TIA-1:TC sites (Figure 4E). Thereupon, excess TC sites resulted in longer lag times and also a lower ThT fluorescence plateau. All ThT signals were corrected for contribution by free ssDNA shown in (Supplementary Figure S4F) These results show that both phase separation and fibril formation of TIA-1 protein can be tuned by both the number of available TIA-1 binding sites and available TIA-1 protein.

### Tandem TIA-1 binding sites in 3′UTR of p53 induce self-association of TIA-1

**Figure 5.**
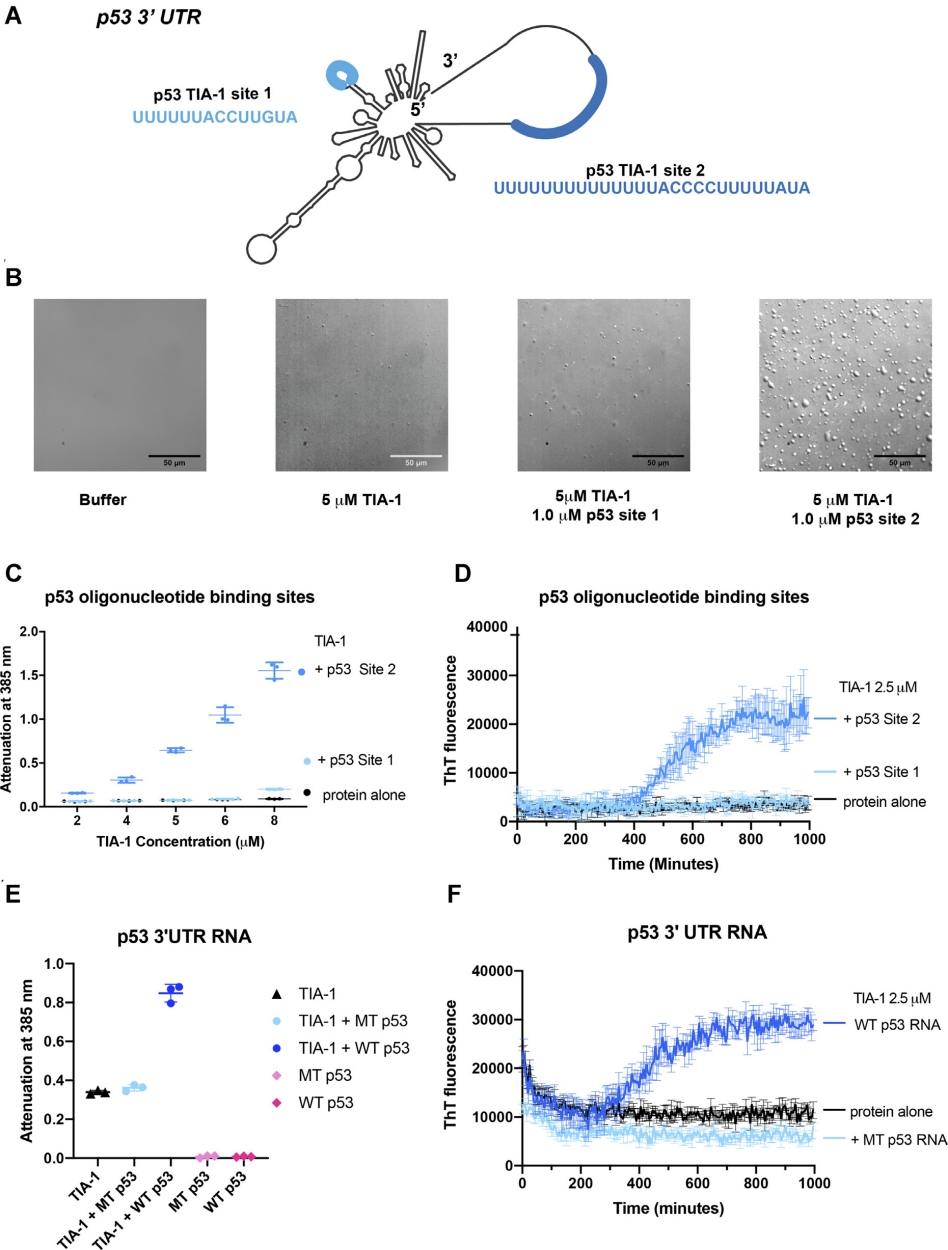
Tandem TIA-1 binding sites in 3′ UTR of p53 mRNA induce phase separation of TIA-1. (**A**) TIA-1 binding sites present in p53 3′UTR. (**B**) DIC microscopy of TIA-1 alone or in presence of p53 ssRNA oligonucleotides. (**C**) Turbidity assay of TIA-1 alone or in the presence of p53 oligonucleotides maintaining 5:1 TIA-1:RNA ratio. (**D**) Thioflavin T assay of TIA-1 (2.5 μM) alone, or in the presence of p53 site 1 or 2 (0.25 μM) monitored at 30 °C. (**E**) Turbidity assay of TIA-1 (2.5 μM) alone or in the presence of p53 3′UTR RNA (0.25 nM) wild type (WT) or with TIA-1 RNA binding sites mutated (MT). (**F**) Thioflavin-T assay of TIA-1 and p53 3′ UTR from E.

Finally, we considered whether RNA representing a native target of TIA-1 would also influence TIA-1 condensation, similarly to our designed model system. One mRNA target of TIA-1 is the well-known genome guardian protein p53 (27). TIA-1 represses translation of p53 mRNA in activated B lymphocytes through binding to two distinct sites identified in the 3′UTR thus controlling protein expression (27). Activation of B cells also induces TIA-1 positive stress granules in which p53 mRNA colocalises (27). These binding sites bear remarkable resemblance to the UC1/TC1 and TC2/TC3 sequences analysed in this study, comprising UC-rich and U-rich elements (Figure 5A). We therefore tested whether these individual sites were sufficient for inducing TIA-1 condensation *in vitro* using oligonucleotides representing either the short 14-nt TIA-1 binding site (p53 site 1) or the longer 27-nt TIA-1 binding site (p53 site 2). P53 site 1 did not induce self-association of TIA-1 either through phase separation or fibril formation as shown by DIC, turbidity and ThT fluorescence assay respectively (Figure 5B-D). This result resembles those found for UC1 and TC1 presumably because this 14-nt site is not long enough to engage two TIA-1 molecules. In contrast, p53 site 2 robustly induced both phase separation of TIA-1 and seeded fibril formation in these same assays. Next we tested whether these TIA-1 binding sites also drove TIA-1 condensation in the context of the p53 3′ UTR. Wild type p53 3′ UTR RNA induced phase separation of TIA-1 in turbidity assay and fibril formation in the ThT assay (Figure 5E and F). In contrast, p53 3′UTR in which both TIA-1 binding sites had been mutated to prevent TIA-1 binding (27) showed no substantial increase in turbidity or ThT fluorescence. Importantly, TIA-1 binding sites occur in single stranded or loop regions of the 3′UTR, and the site mutations did not change predicted folding of the RNA. These experiments demonstrate that native TIA-1 tandem binding sites in a 3′ UTR can induce TIA-1 protein condensation.

## DISCUSSION

LLPS by RNA binding proteins is key to the formation, maintenance and dissolution of biomolecular condensates such as stress granules. While phase separation is often mediated by the intrinsically disordered low-complexity domains of RBPs, the role of RNA is clearly vital in this process and thus also to potential progression to irreversible aggregates. RNA directly contributes to biomolecular condensates both through direct RNA-RNA interactions, as well as by enhancing and buffering the self-association properties of RNA binding proteins (6). Exploring the basic principles and interplay between RBPs and RNA is thus essential for gaining fundamental insight into how biomolecular condensates form and can be regulated. Here we used a series of designed and native oligonucleotide binding sites of the stress granule protein TIA-1 and showed that nucleic acid promotes TIA-1 phase separation in a sequence specific and multisite dependent manner and also promotes formation of amyloid like fibrils.

Our study explored the mechanism by which TIA-1 self-association is enhanced by RNA. We established conditions under which TIA-1 does not undergo phase separation on its own but can be triggered to do so by the addition of oligonucleotide. This allowed us to observe that single site RNA or DNA binding by TIA-1 does not increase its propensity to phase separate or form amyloid-like fibrils. In contrast, multisite oligonucleotides induced a robust enhancement of TIA-1 condensation. Since TIA-1 RRM23 domains alone did not phase separate upon addition of oligonucleotide, it is concluded that the PrLD interactions of the bound TIA-1 molecules facilitate the condensation. This is in agreement with the general view that multivalent RNA can act as a scaffold for biomolecular condensates, but is one of only a few demonstrations of the synergy between PrLD and RNA interactions underlying LLPS. Similar observations have been made for the Whi3 protein (16,17), FUS (13,41) and chimeric PTB-RRM_4_-PrLD proteins (21,22) in the presence of RNA with specific or non-specific target sites.

In addition to acting as a scaffold nucleating LLPS, RNA binding by TIA-1 may also act to induce a conformational change that favours LLPS. Under the conditions of our experiments SAXS data indicated a conformational change upon oligonucleotide binding consistent with an extension of the PrLD, though not resulting in an overall increase of flexibility of TIA-1. The PrLD is likely flexible and available for inter-molecular interactions both before and after TIA-1 binds RNA, but there may be a shift in equilibrium to more elongated states upon binding. Thus, in the case of TIA-1, there may be a combined effect of TIA-1 scaffolding and conformational change upon RNA binding in the triggering of condensation. Such a combination of effects has recently been reported for the SG initiating protein G3BP. In the case of G3BP it was shown that conformational change upon RNA binding drives condensation and also observed that longer RNA, but not shorter RNA (60 nucleotides) were efficient in inducing G3BP assemblies (18,19). In the case of G3BP, however, a PrLD was not present, but rather a dimerization domain was proposed to act alongside RNA-binding domains to promote clustering.

In this study, the same multisite nucleic acids that enhanced phase separation of TIA-1 also, over a number of hours, enhanced the formation of irreversible amyloid like fibrils. Nucleic acid induced fibril formation reflected classical kinetics, showing a lag phase, an elongation phase that could be delayed by later nucleation by oligonucleotide, and an eventual plateau of fibril formation (48). As such, multisite binding appears to act as a nucleation event *in vitro* allowing the formation of β-sheet conformation and subsequent growth phase of PrLD amyloid fibril growth to commence. The similar effect of multisite nucleic acids on both phase separation and fibril formation, together with the coexistence of phase separated droplets and fibrils suggest that fibrils could form through a maturation from phase separated droplets as has been observed for PrLD proteins FUS (10), hnRNPA1 (49) and TDP-43 PrLD (50). But it is also possible that multisite nucleic acids enhance two separate assembly processes. Our study is consistent with the understanding that protein-RNA interactions can result in a number of physical states from dynamic liquid condensates, reversible hydrogels to solid reversible and irreversible fibrillar aggregates. The potential aberrant phase behaviour of PrLD containing stress granule proteins is associated with neurotoxic aggregates formed in neurodegenerative diseases (9,51) Together this suggests that cellular mechanisms or therapeutic approaches that modulate phase separation will also have a direct effect on the formation of neurotoxic aggregate.

Having established that either RNA or DNA with five TIA-1 binding sites readily induces LLPS and propagation to fibril formation, we explored whether the number of binding sites and their proximity to one another impacted this. We found that as few as 2–3 tandem binding sites were sufficient to induce robust phase separation and fibril formation by TIA-1 and this was not enhanced by longer oligonucleotides with additional concatenated binding sites. This has interesting implications for the way in which TIA-1 undergoes phase separation and sequesters mRNA to SGs. TIA-1 often binds mRNA in clusters of binding sites in 3′UTR to regulate translation (24) and one such cluster occurs in the 3′ UTR of p53 (27). We showed that the 3′ UTR of p53 induces TIA-1 condensation via this cluster of TIA-1 sites. Interestingly, the tandem ‘site 2’ effectively induced TIA-1 condensation whereas the individual ‘site 1’ did not, elegantly mirroring our results with our designed nucleic acids. Thus, just two native tandem high affinity TIA-1 binding sites in mRNA 3′ UTRs may act as instigators of phase separation, contributing to SG formation.

Our study unexpectedly revealed that condensation by TIA-1 can also be driven by more promiscuous interactions with RNA. Although our study investigated the effect of high affinity, 10-nt sequence specific TIA-1 binding sites, our results with the complementary sequence to TC5, also suggest that multiple suboptimal TIA-1 binding sites are sufficient to induce phase separation. Despite having no intended TIA-1 binding sites, ‘TC5 complement’ showed effective induction of phase separation and fibril formation *in vitro* potentially through four coincidental ATT motifs (24). Initial stress granule formation in cells is driven by the availability of recently exposed mRNA, and subsequent binding of stress granule proteins. Such short ‘suboptimal’ as well as higher affinity motifs are likely to exist in the newly exposed mRNAs, effectively sequestering TIA-1 and inducing local phase separation. Phase separation by other RNA binding SG proteins such as G3BP1 and hnRNPA1 is also enhanced by RNA with degenerate biased sequence composition rather than strict linear sequence motifs to sequester the heterogeneous mRNA to SGs. Specific, high affinity binding sites, and degenerate, lower affinity binding sites bound by RNA binding proteins represent two types of RNA interactions which, in the case of TIA-1 and hnRNPA1 can occur for the same protein (52). The different role between short high affinity RNA ‘seeds’ and low affinity degenerate motifs in stress granule formation remains poorly understood.

TIA-1 condensation in our study was acutely sensitive to the ratio and concentration of high affinity tandem TIA-1 binding sites. Optimal phase separation enhancement was achieved when TIA-1 was combined in a 1:1 ratio with TIA-1 binding sites. In contrast, excess TIA-1 binding sites acted as a competitive inhibitor of phase separation and fibril formation. This is somewhat reminiscence of the ‘re-entrant’ behaviour of Arg-rich peptides with homopolymer RNA in which attractive forces enhance condensation up to a point at which repulsive forces begin to predominate (47,53). Such a buffering effect is considered to be the phenomenon underlying the solubilisation of RBPs in the presence of excess RNA (15). Using relatively short oligonucleotides with defined TIA-1 binding sites we were able to precisely define the dependence of protein: RNA binding ratios on TIA-1 propensity to initiate LPPS and fibril formation. This demonstration of fine tuning suggests that the system may be amenable to modulation by agents that interact with TIA-1. In particular, our data suggest that modulation of TIA-1 binding by short oligonucleotide mimetics or small molecules targeting the RNA binding site could be used to enhance the dissolution of SGs (14,54–56).

In summary, we have shown that multiple TIA-1 nucleic acid binding sites induce aggregation of TIA-1 both through LLPS and fibril formation in a sequence specific manner under conditions of excess protein. These results demonstrate the ability of oligonucleotide to scaffold the TIA-1 protein thus facilitating further protein-protein interactions by the intrinsically disordered PrLD. Excess nucleic acid sites can slow and partially inhibit TIA-1 condensation and fibril formation most likely by diluting local protein concentration. Thus, RNA binding by TIA-1 can both control the self-association potential of the PrLD of TIA-1 as well as sequester mRNAs to SGs.

## DATA AVAILABILITY

SAXS data have been deposited in the Small Angle Scattering Biological Data Bank (SASBDB) under the accession numbers: SASDJK5: TIA-1 isoform p40 (TIA-1) bound to TC1 DNA; SASDJL5: TIA-1 isoform p40 (TIA-1) bound to UC1 RNA; SASDJM5: TIA-1 isoform p40 (TIA-1 APO).

## Supplementary Material

gkab080_Supplemental_File

## Notes

Present address: Saboora Waris, Shaheed Zulfiqar Ali Bhutto Medical University (SZABMU), Pakistan Institute of Medical Sciences, G-8/3, Islamabad 44000, Pakistan.
