# Supplementary material for: Tandem RNA binding sites induce self-association of the stress granule marker protein TIA-1
Source: Nucleic Acids Res. 2021 Feb 24;49(5):2403–17. doi: 10.1093/nar/gkab080 (PMC7969032; doi:10.1093/nar/gkab080)
Supplement: gkab080_Supplemental_File [file gkab080_supplemental_file.pdf]

SUPPLEMENTARY DATA

Tandem RNA binding sites induce self-association of the stress granule marker protein TIA-1

Fionna E. Loughlin, Danella L. West, Menachem J. Gunzburg, Saboora Waris, Simon A. Crawford, Matthew C.J. Wilce and Jacqueline A. Wilce

Supplementary Figure 1. Sequence specific multisite RNA and DNA induces liquid-liquid phase separation of TIA-1

Supplementary Figure 2. Nucleic acid containing multiple TIA-1 binding sites induced fibril formation of TIA-1.

Supplementary Figure 3. SAXS analysis shows slight elongation of TIA-1 on binding ssRNA UC1 and ssDNA TC1 (Active voice caption)

Supplementary Figure 4. The ratio of TIA-1 protein:tandem binding sites finely tunes liquid-liquid phase separation and fibril formation.

Supplementary Table 1. Nucleic acids used in this study

Supplementary Table 2. Absorption values in C_{sat} measurements from Figure 1

Supplementary Table 3. Absorption values in C_{sat} measurements from Figure 4

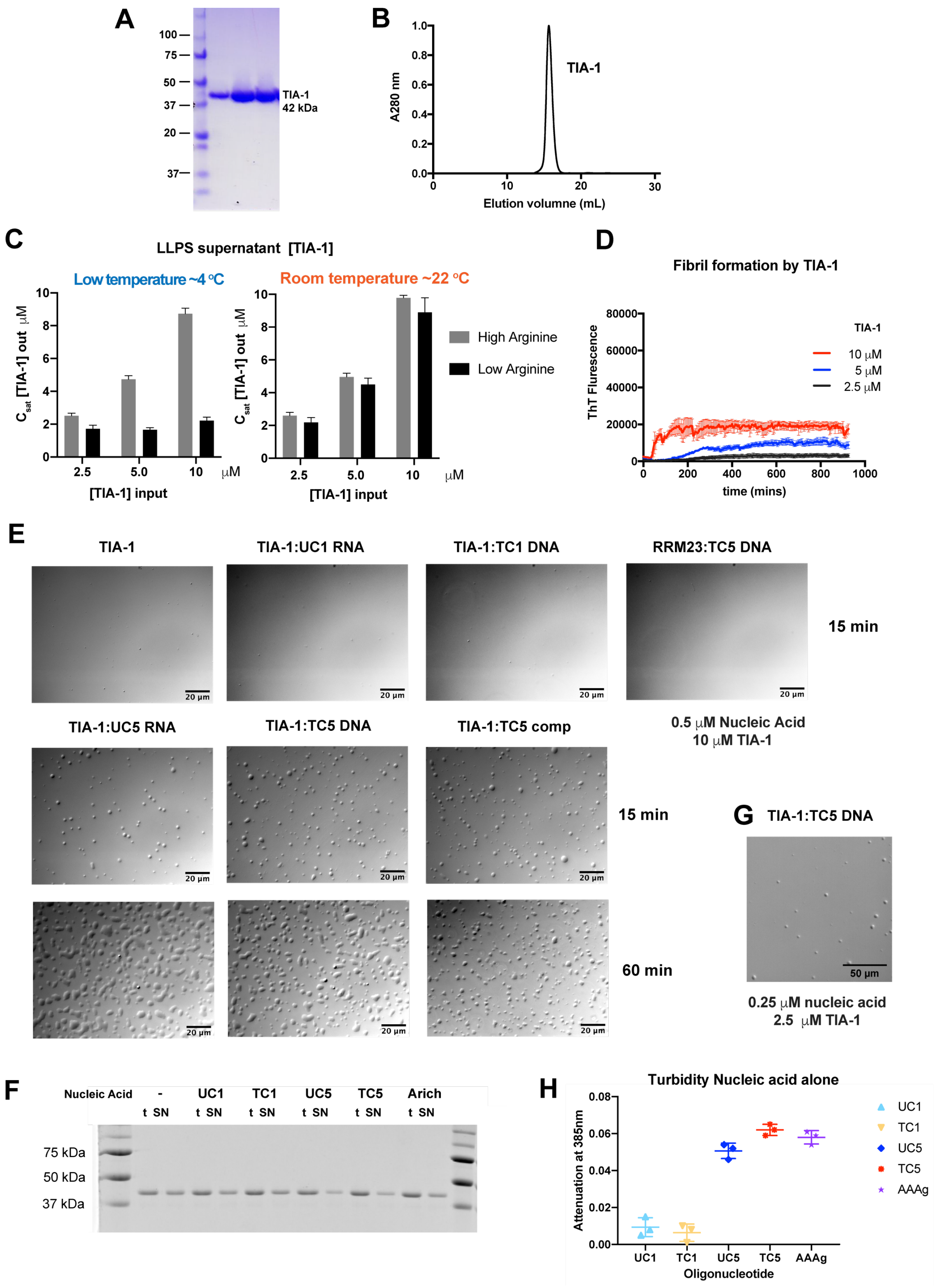

Supplementary Figure 1. Sequence specific multisite RNA and DNA induces liquid-liquid phase separation of TIA-1. A) SDS-PAGE of purified TIA-1, B) Size exclusion of TIA-1, C) The concentration of TIA-1 protein remaining dispersed in supernatant solution (C_{sat}) after phase separation at 4 °C or room temperature. D) Thioflavin T fluorescence monitoring aggregation of TIA-1 protein at 30 °C. E) DIC microscopy of TIA-1 (10 µM) alone or in the presence of nucleic acids (0.5 µM). F) SDS-PAGE analysis of TIA-1 protein dispersed in

supernatant solution (t: total sample, SN: supernatant after spin) shows depletion of TIA-1 protein in supernatant. G) DIC microscopy of TIA-1 (2.5 μM) in the presence of TC5 (0.25 μM). H) Turbidity measurements of nucleic acid alone. Assays show 3 replicates and error bars representing S.D.

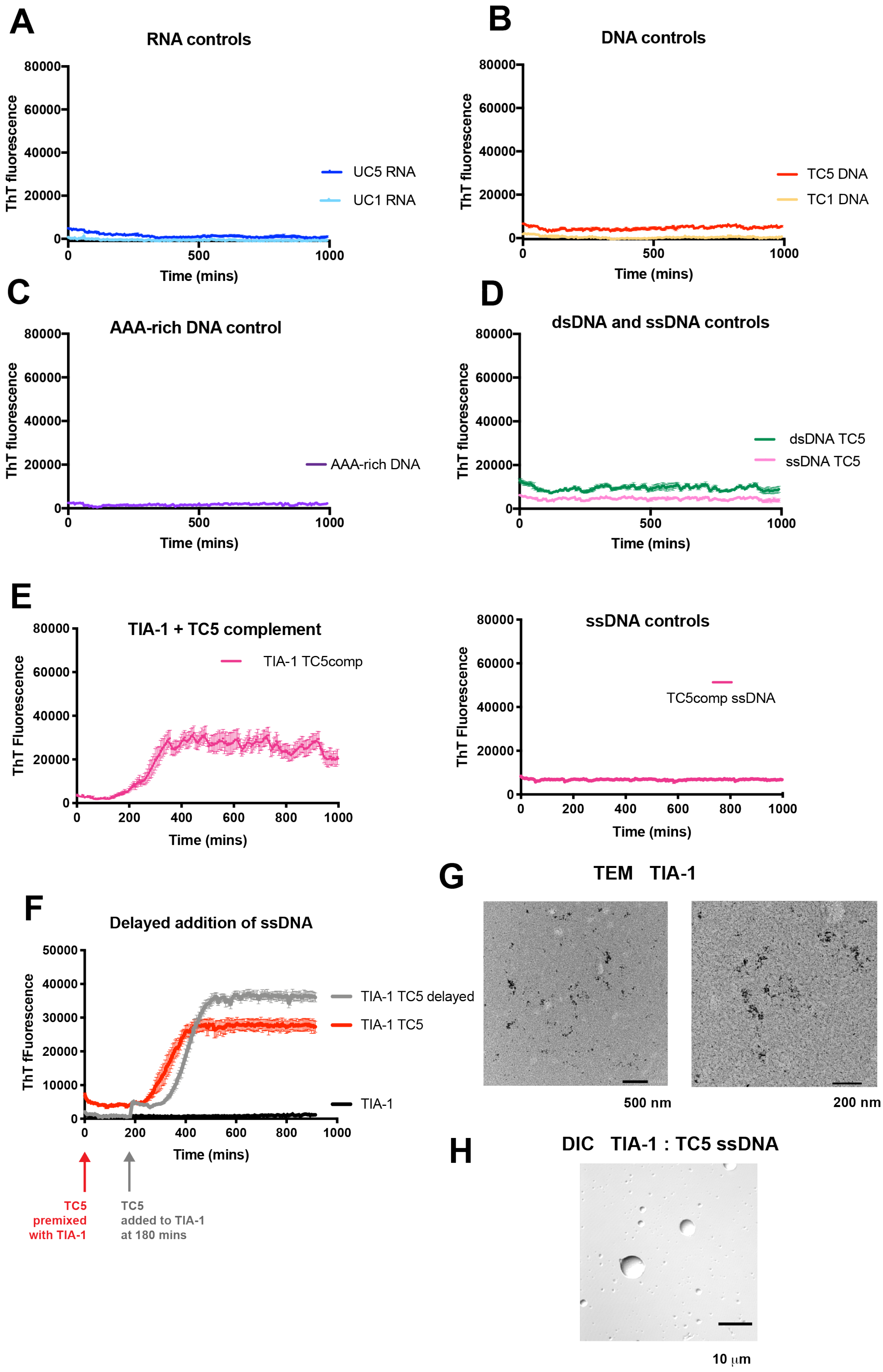
A
RNA controls
ThT fluorescence
80000
60000
40000
20000
0
UC5 RNA
UC1 RNA
0
500
1000
Time (mins)
B
DNA controls
ThT fluorescence
80000
60000
40000
20000
0
TC5 DNA
TC1 DNA
0
500
1000
Time (mins)
C
AAA-rich DNA control
ThT fluorescence
80000
60000
40000
20000
0
AAA-rich DNA
0
500
1000
Time (mins)
D
dsDNA and ssDNA controls
ThT fluorescence
80000
60000
40000
20000
0
dsDNA TC5
ssDNA TC5
0
500
1000
Time (mins)
E
TIA-1 + TC5 complement
ThT Fluorescence
80000
60000
40000
20000
0
TIA-1 TC5comp
0
200
400
600
800
1000
Time (mins)
ssDNA controls
ThT Fluorescence
80000
60000
40000
20000
0
TC5comp ssDNA
0
200
400
600
800
1000
Time (mins)
F
Delayed addition of ssDNA
ThT fFuorescence
50000
40000
30000
20000
10000
0
TIA-1 TC5 delayed
TIA-1 TC5
TIA-1
0
200
400
600
800
1000
Time (mins)
TC5
premixed
with TIA-1
TC5
added to TIA-1
at 180 mins
G
TEM TIA-1
500 nm
200 nm
H
DIC TIA-1 : TC5 ssDNA
10 μm

Supplementary Figure 2. Nucleic acid containing multiple TIA-1 binding sites induced fibril formation of TIA-1. A-D) ThT fluorescence measurements of nucleic acid alone used in Figure 1 A-D. A) ssRNA UC1 (*light blue*) and UC5 (*blue*), B) ssDNA TC1 (*yellow*) and TC5 (*red*), C) ss AAA-rich DNA (*purple*), D) dsDNA dsTC5 (*green*) and ssDNA TC5 (*pink*). E) ThT fluorescence measurement of TIA-1 in presence of ssDNA TC5 complement (left) and ssDNA TC5 complement alone (right). F) Comparison of TIA-1 pre-mixed with TC5 (*red*) or adding TC5 after 200 minutes (*grey*). G) Representative TEM images TIA-1 alone showing no fibril formation by TIA-1 at 2.5 μM in 20 mM HEPES pH 7.2, 50 mM NaCl, 40 mM Arginine. H) DIC microscopy of TIA-1 TC5 ssDNA from ThT assay after ~ 10 hours showing the presence of phase separated liquid droplets.

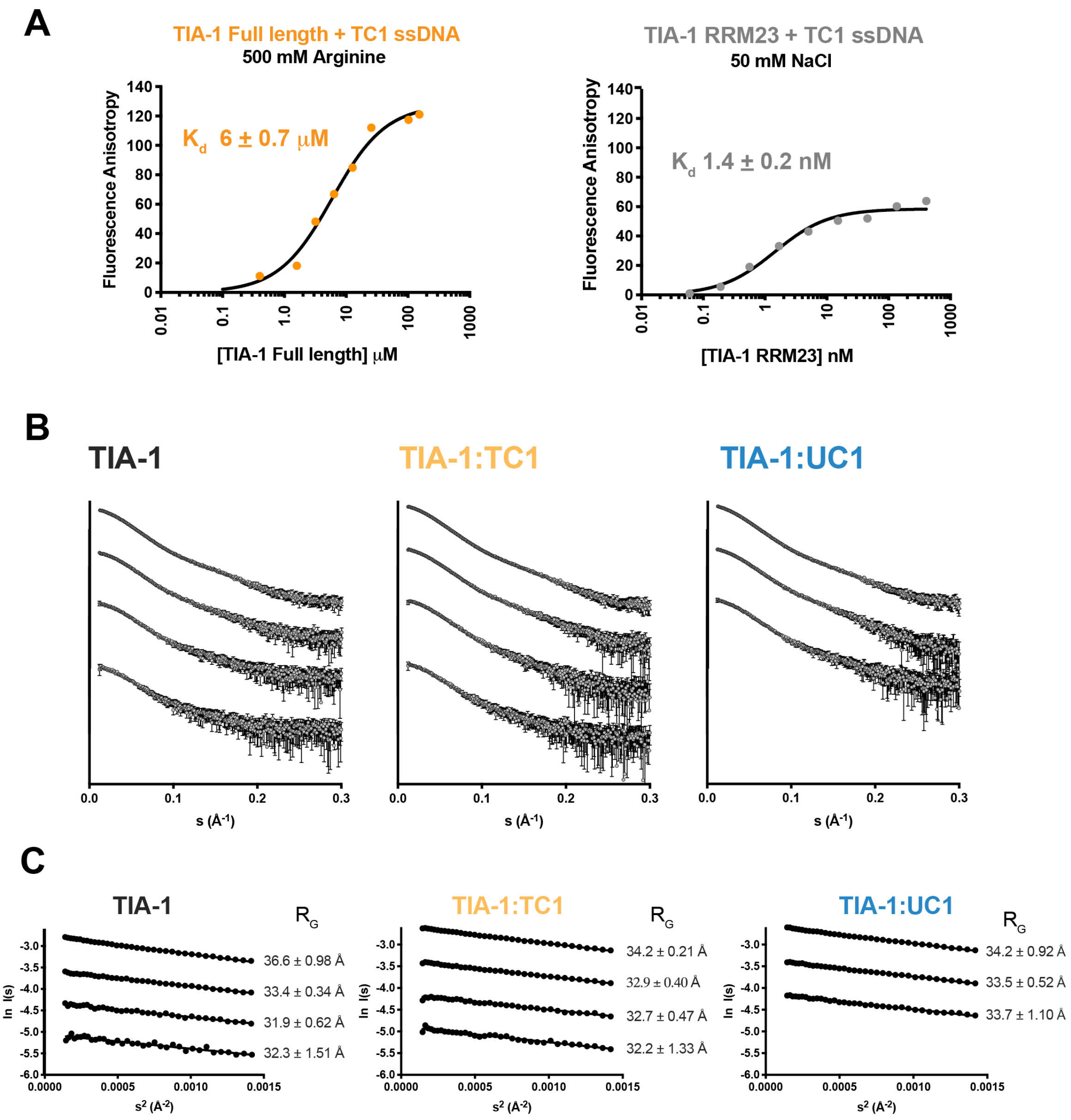

Supplementary Figure 3. SAXS analysis shows slight conformational change of TIA-1 on binding ssRNA UC1 and ssDNA TC1. A) Fluorescence anisotropy of TIA-1 full length binding to Fl-TC1 in size exclusion buffer (20 mM Sodium phosphate, 60 mM KCl, 0.5 M Arginine-HCl, 1mM $MgCl_2$, 2 mM DTT, 0.5 mM EDTA, pH 7) compared to TIA-1 RRM23 binding to the same oligonucleotide in aggregation buffer (20 mM HEPES, 50 mM NaCl 1 mM DTT). B) Experimental scattering profiles of TIA-1 alone and in complex with ssDNA TC1 and ssRNA UC1 obtained by plotting the log of scattering intensity [log I(s)] as a function of forward scattering vector in the units of Å $^{-1}$ with grey spheres and black error bars representing the ± SD. C) Gunier Plots of TIA-1 and nucleic acid complexes and Rg over concentrations ranges 0.37, 0.73, 1.5, 2.9 mg/mL

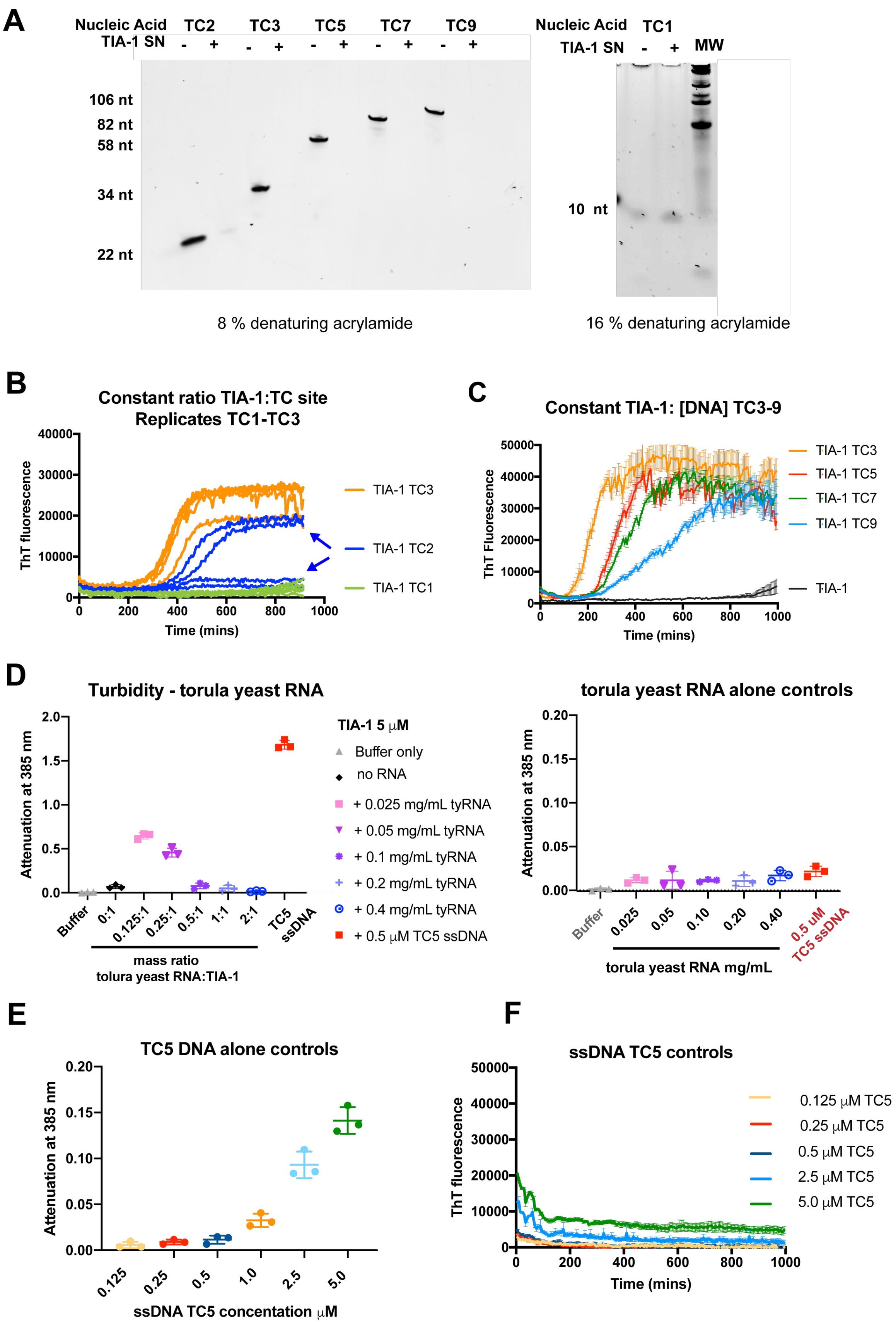

A
Nucleic Acid TC2 TC3 TC5 TC7 TC9
TIA-1 SN - + - + - + - + - +
106 nt
82 nt
58 nt
34 nt
22 nt
8 % denaturing acrylamide
Nucleic Acid TC1
TIA-1 SN - + MW
10 nt
16 % denaturing acrylamide
B
Constant ratio TIA-1:TC site
Replicates TC1-TC3
ThT fluorescence
Time (mins)
TIA-1 TC3
TIA-1 TC2
TIA-1 TC1
C
Constant TIA-1: [DNA] TC3-9
ThT Fluorescence
Time (mins)
TIA-1 TC3
TIA-1 TC5
TIA-1 TC7
TIA-1 TC9
TIA-1
D
Turbidity - torula yeast RNA
Attenuation at 385 nm
Buffer
0:1
0.125:1
0.25:1
0.5:1
1:1
2:1
TC5 ssDNA
mass ratio
tolura yeast RNA:TIA-1
TIA-1 5 μM
Buffer only
no RNA
+ 0.025 mg/mL tyRNA
+ 0.05 mg/mL tyRNA
+ 0.1 mg/mL tyRNA
+ 0.2 mg/mL tyRNA
+ 0.4 mg/mL tyRNA
+ 0.5 μM TC5 ssDNA
torula yeast RNA alone controls
Attenuation at 385 nm
Buffer
0.025
0.05
0.10
0.20
0.40
0.5 uM TC5 ssDNA
torula yeast RNA mg/mL
E
TC5 DNA alone controls
Attenuation at 385 nm
0.125
0.25
0.5
1.0
2.5
5.0
ssDNA TC5 concentation μM
F
ssDNA TC5 controls
ThT fluorescence
Time (mins)
0.125 μM TC5
0.25 μM TC5
0.5 μM TC5
2.5 μM TC5
5.0 μM TC5

Supplementary Figure 4. The ratio of TIA-1 protein: binding sites finely tunes liquid-liquid phase separation and fibril formation. A) Denaturing PAGE gels showing ssDNA in input (-) and in supernatant (-) from C_{sat} experiments. B&C) ThT assay of TIA-1 with ssDNA at constant TC binding site concentrations: B) TC1-3 showing individual replicates. C) ThT assay of TIA-1 in presence of TC3-TC9 at the same molar ratio. D) Turbidity assay of TIA-1 (5 μM) with increasing amounts of tolura yeast RNA or TC5 ssDNA (0.5 μM) *left* and controls for nucleic acid alone, *right*. E&F) Control of ssDNA alone: E) ThT assay of ssDNA alone for increasing TC5 concentration. F) Turbidity of ssDNA alone for increasing TC5 concentration.

Supplementary Table 1. Nucleic acids used in this study

Nucleic acid	nt	sequence	# TC sites
		DNA	
**TC1**	10	TTTTTACTCC	1
**TC1.5**	15	TTTTTTTTTTACTCC	1
**TC2**	22	TTTTTACTCCAATTTTTACTCC	2
**TC3**	34	TTTTTACTCCAATTTTTACTCCAATTTTTACTCC	3
**TC5**	58	TTTTTACTCCAATTTTTACTCCAATTTTTACTCCAATTTTTACTCCAATTTTTACTCC	5
**TC7**	82	TTTTTACTCCAATTTTTACTCCAATTTTTACTCCAATTTTTACTCCAATTTTTACTCCAA<br>TTTTTACTCCAATTTTTACTCC	7
**TC9**	106	TTTTTACTCCAATTTTTACTCCAATTTTTACTCCAATTTTTACTCCAATTTTTACTCCAA<br>TTTTTACTCCAATTTTTACTCCAATTTTTACTCCAATTTTTACTCC	9
**Arich**		AAAAAAAAAAGAGAAAAAAAAGAGAAAAAAAAAAGAGAAAAAAAAAAGAGAAAAAAAA	0
**dsTC5**	58 x 2	TTTTTACTCCAATTTTTACTCCAATTTTTACTCCAATTTTTACTCCAATTTTTACTCC<br>AAAAATGAGGTTAAAAATGAGGTTAAAAATGAGGTTAAAAATGAGGTTAAAAATGAGG	5
**TC5 comp**		AAAAATGAGGTTAAAAATGAGGTTAAAAATGAGGTTAAAAATGAGGTTAAAAATGAGG	
		RNA	
**UC1**	10	UUUUUACUCC	1
**UC5**	58	UUUUUACUCCAAUUUUUACUCCAAUUUUUACUCCAAUUUUUACUCCAAUUUUUACUCC	5
**P53 3' UTR site 1**	14	UUUUUUACCUUGUA	1
**P53 3' UTR site 2**	27	UUUUUUUUUUUUUUACCCCUUUUUAUA	2
**P53 3' UTR wt**	326	Gggucccuucugcugcc**uuuuuuaccuug**uagcuag<br>ggcucagccccucucugaguagugguuccuggcccaaguuggggaauagguugauaguu<br>gucaggucucugcuggcccagcgaaauucuauccagccaguuguuggacccuggcaccua<br>caaugaaaucucacccuaccccacacccuguaagauucuaucuugggcccucauaggguc<br>cauauccuccagggccuacuuuccuuccauucugcaaagccugucugcauuuauccaccc<br>cccacccugucucccuc**uuuuuuuuuuuuuuaccccuuuuu**auauaucaa	
**P53 3'UTR mut**	326	Gggucccuucugcugcc**uauguaaccuag**uagcuag<br>ggcucagccccucucugaguagugguuccuggcccaaguuggggaauagguugauaguu<br>gucaggucucugcuggcccagcgaaauucuauccagccaguuguuggacccuggcaccua<br>caaugaaaucucacccuaccccacacccuguaagauucuaucuugggcccucauaggguc<br>cauauccuccagggccuacuuuccuuccauucugcaaagccugucugcauuuauccaccc<br>cccacccugucucccuc**uauguauguauguaaccccugauu**auauaucaa	

		DNA for preparing 3' UTR RNA templates	
**P53 3'UTR wt DNA template**		GCTTAATACGACTCACTATAGggtcccttctgctgcc**tttttttaccttg**tagctag<br>ggctcagcccctctctgagtagtggttcctggcccaagttggggaataggttgatagtt<br>gtcaggtctctgctggcccagcgaaattctatccagccagttgttggaccctggcaccta<br>caatgaaatctcaccctaccccacaccctgtaagattctatcttgggccctcatagggtc<br>catatcctccagggcctactttccttccattctgcaaagcctgtctgcatttatccaccc<br>cccaccctgtctccctc	
**P53 3' UTR mut DNA template**		GCTTAATACGACTCACTATAGggtcccttctgctgcc**tatgtaacctag**tagctag<br>ggctcagcccctctctgagtagtggttcctggcccaagttggggaataggttgatagtt<br>gtcaggtctctgctggcccagcgaaattctatccagccagttgttggaccctggcaccta<br>caatgaaatctcaccctaccccacaccctgtaagattctatcttgggccctcatagggtc<br>catatcctccagggcctactttccttccattctgcaaagcctgtctgcatttatccaccc<br>cccaccctgtctccctc**tatgtatgtatgtaacccctgatt**atatatcaa	
**P53 Fwd primer**		GCTTAATACGACTCACTATAG	
**P53 wt Rev primer**		ttgatatataaaaaggggtaaaaaaaaaaaaaagagggagacagggtggg	
**P53 mut Rev primer**		ttgatatataatcaggggttac	

Supplementary Table 2. Absorption values in C_{sat} measurements from Figure 1

Sample	A_{280}	$A_{260/280}$	Corrected A_{280}
TIA-1	0.69 0.66 0.80	0.64	
TIA-1 + UC1	0.68 0.62 0.69	0.68	
TIA-1 + TC1	0.64 0.64 0.63	0.67	
TIA-1 + UC5	0.17 0.14 0.17	0.70	
TIA-1 + TC5	0.18 0.21 0.19	0.68	
TIA-1 + AAg*	0.73 0.80 0.72	1.08	0.54 0.51 0.51
TIA-1 + dsTC5*	0.77 0.71. 0.77	1.03	0.59 0.61 0.58
TIA-1 + TC5compl	0.29 0.29 0.33	0.76	

* Corrected for contribution from nucleic acid remaining in the supernatant

Supplementary Table 3. Absorption values in C_{sat} measurements from Figure 4

Sample	A_{280}	$A_{260/280}$	Corrected A_{280}
TIA-1	0.70 0.76. 0.73	0.62	
TIA-1 + TC1*	0.87 0.88. 0.88	0.72	0.71 0.72 0.72
TIA-1 + TC2	0.38 0.41 0.46	0.67	
TIA-1 + TC3	0.36 0.38 0.38	0.68	
TIA-1 + TC5	0.44 0.45 0.49	0.63	
TIA-1 + TC7	0.37 0.37. 0.41	0.68	
TIA-1 + TC9	0.39 0.43. 0.39	0.65	

* Corrected for contribution from nucleic acid remaining in the supernatant
